# Research progress of 3D-printed anti-infective bone tissue engineering scaffolds based on triply periodic minimal surface structures

**DOI:** 10.1063/5.0334789

**Published:** 2026-06-05

**Authors:** Peijie Zhao, Yafeng Zhang, Zewen Qiao

**Affiliations:** 1 Shantou Central Hospital, Shantou 515000, China; 2 Ningxia Medical University, Yinchuan 750000, China; 3 General Hospital of Ningxia Medical University, Yinchuan 750000, China

## Abstract

Bone defect repair remains a significant clinical challenge, necessitating scaffold materials that combine excellent mechanical properties, bioactivity, and anti-infective capabilities, which are central to bone tissue engineering. Triply periodic minimal surface (TPMS) structures have garnered considerable attention due to their superior mechanical and biological characteristics, demonstrating great potential in the design of bone repair scaffolds. This review summarizes the latest advances in three-dimensional printed anti-infective tissue engineering scaffolds based on TPMS structures within the field of bone regeneration. It highlights the design advantages of TPMS architectures, the performance of composite materials such as polylactic acid/magnesium titanate, and the antimicrobial mechanisms of these scaffolds. Furthermore, the synergistic effects of promoting osteogenesis and combating infection are analyzed. By systematically collating current research findings, this article aims to provide a theoretical foundation and guidance for the development of next-generation multifunctional bone repair materials.

## I. INTRODUCTION

Bone tissue engineering (BTE) has emerged as a transformative strategy to address the limitations inherent in traditional bone grafting methods, such as autografts and allografts, which are often constrained by donor site morbidity, limited availability, immune rejection, and risk of infection.^1^ The fundamental goal of BTE is to develop biomimetic scaffolds that provide a three-dimensional (3D) microenvironment conducive to cell adhesion, proliferation, differentiation, and ultimately new bone formation, thereby facilitating the repair of critical-sized bone defects that cannot heal spontaneously.^2^ These scaffolds must fulfill multifaceted criteria: they should possess mechanical properties compatible with native bone to provide temporary structural support; exhibit excellent biocompatibility and bioactivity to promote osteogenesis; and crucially, incorporate functionalities to prevent or mitigate infection during the early post-implantation period, which is a pivotal determinant of clinical success.

The design of bone scaffolds has progressively evolved from simple porous constructs to sophisticated architectures that closely emulate the hierarchical and anisotropic nature of natural bone.^3^ Among these, triply periodic minimal surfaces (TPMSs) have garnered significant attention due to their mathematically defined, continuous, and highly interconnected porous structures. TPMS-based scaffolds exhibit high specific surface areas, smooth curvature, and tunable mechanical properties, enabling them to effectively mimic the trabecular microarchitecture of cancellous bone. This topology facilitates efficient nutrient and waste transport, supports cell migration, and provides mechanical cues essential for osteogenic differentiation.^4^ The inherent periodicity and minimal surface characteristics of TPMS structures also contribute to uniform stress distribution, reducing stress concentrations that could compromise scaffold integrity under physiological loading conditions.

Advancements in additive manufacturing, particularly 3D printing technologies such as fused deposition modeling (FDM), selective laser sintering (SLS), and stereolithography, have revolutionized the fabrication of TPMS scaffolds. These techniques enable precise control over scaffold geometry, pore size, porosity, and interconnectivity, allowing for patient-specific customization and reproducibility.^5^ For instance, the integration of TPMS geometries with biodegradable polymers like polylactic acid (PLA) and composites incorporating bioactive fillers such as graphene oxide (GO) or magnesium titanate (MgTiO_3_) has demonstrated enhanced mechanical strength, osteoconductivity, and bioactivity. Moreover, the ability to modulate scaffold architecture at the micro- and nanoscale facilitates the creation of biomimetic environments that promote cellular responses critical for bone regeneration.^6^

In parallel, the incorporation of bioactive molecules and ions into scaffolds has been extensively explored to augment their osteoinductive and antimicrobial capabilities. Growth factors such as vascular endothelial growth factor (VEGF) and bone morphogenetic proteins (BMPs) have been loaded into hydrogel-based or composite scaffolds to enhance angiogenesis and osteogenesis, thereby accelerating bone repair.^7,8^ However, challenges related to the stability, controlled release, and potential side effects of these proteins have spurred interest in alternative strategies. The use of bioactive ions (e.g., Mg^2+^, Sr^2+^, Zn^2+^, and CeO_2_ nanoparticles) and small molecules has shown promise in modulating the local microenvironment to favor bone regeneration while simultaneously exerting antibacterial effects. For example, magnesium and strontium ions released from composite hydrogels can synergistically promote mesenchymal stem cell (MSC) proliferation and differentiation, mimicking the natural hematoma environment during bone healing.^9^ Similarly, cerium oxide-reinforced bioactive glass (BG) scaffolds have demonstrated sequential therapeutic effects by alleviating oxidative stress and enhancing osteogenesis.

Infection remains a formidable obstacle in bone repair, often leading to implant failure and necessitating revision surgeries. Therefore, the development of scaffolds with intrinsic antibacterial properties is critical. Strategies include the incorporation of antimicrobial agents such as copper-doped layered double hydroxides, chitosan-based composites, and antimicrobial peptides, which can provide localized infection control without systemic toxicity. Additionally, the use of multifunctional scaffolds capable of photothermal therapy or mild hyperthermia has been investigated to eradicate bacteria while promoting osteogenesis and angiogenesis.^10^ The integration of such functionalities within TPMS-based scaffolds fabricated via 3D printing holds significant potential for creating next-generation bone substitutes that address both mechanical and biological challenges.

Despite these advances, several challenges persist in the clinical translation of TPMS-structured, 3D-printed bone scaffolds with antibacterial functions.^11^ These include optimizing the balance between porosity and mechanical strength to withstand physiological loads, ensuring controlled and sustained release of bioactive agents, achieving uniform cell seeding and vascularization within complex architectures, and scaling up manufacturing processes while maintaining quality and reproducibility. Furthermore, the long-term biocompatibility and degradation behavior of composite materials incorporating metallic ions or nanoparticles require thorough investigation to preclude adverse effects.^12^

In summary, the convergence of TPMS-based scaffold design, advanced 3D printing technologies, and multifunctional biomaterials incorporating osteogenic and antibacterial components represents a promising frontier in bone tissue engineering.^13,14^ By emulating the native bone microenvironment and addressing infection risks, these innovative scaffolds have the potential to significantly improve outcomes in the repair of critical-sized bone defects.^15^ This review systematically delineates the principles underlying the design and fabrication of TPMS-structured 3D-printed scaffolds, examines the roles of composite materials and bioactive agents in enhancing scaffold performance, and discusses the biological effects and future challenges in this rapidly evolving interdisciplinary field (Table I).

**TABLE I.** Summary of materials and manufacturing technologies for TPMS-structured bone tissue engineering scaffolds.

Category	Specific material/technique	Composition system	Advantages	Limitations	References
3D printing manufacturing techniques	FFF/FDM	Fused filament fabrication/fused deposition modeling, layer-by-layer deposition of molten thermoplastic filaments	1. Low cost and simple operation process; 2. Excellent printability for biodegradable polymers such as PLA; 3. Capable of fabricating complex TPMS architectures with controllable porosity; 4. Suitable for polymer-ceramic composite scaffold preparation	1. Relatively low printing accuracy and limited structural fidelity; 2. Nozzle clogging easily caused by ceramic fillers; 3. Insufficient interlayer bonding weakens overall mechanical properties	1, 16–19
	SLA/DLP	Stereolithography/digital light processing, UV-cured additive manufacturing	1. High printing resolution and superior micro/nano structural fidelity; 2. Smooth scaffold surface with low roughness; 3. Ideal for high-precision TPMS structure fabrication	1. Limited material selection, mostly photosensitive polymers; 2. Additional biocompatibility verification required for cured resin products	20
	LPBF	Laser powder bed fusion, powder-based laser sintering/melting	1. Suitable for metallic and ceramic biomaterials; 2. Outstanding mechanical properties, compatible with load-bearing bone repair	1. High equipment and processing costs; 2. Complex post-processing procedures and high technical threshold	20
Polymer matrix materials	PLA	Pure polylactic acid	1. Good biodegradability and cytocompatibility; 2. Outstanding FFF printability; 3. Mature clinical application history and reliable biosafety	1. Strong hydrophobicity, unfavorable for cell adhesion and protein adsorption; 2. Insufficient mechanical strength for heavy load-bearing sites; 3. Acidic degradation byproducts may trigger local inflammation; 4. No bioactivity or intrinsic antibacterial property	21, 22
Composite ceramic phases	MgTiO_3_	PLA/MgTiO_3_ composite	1. Significantly improves compressive strength and modulus of PLA matrix; 2. Enhances thermal stability (increases thermal decomposition temperature); 3. Reduces water contact angle and improves surface hydrophilicity; 4. Releases Mg^2+^ to promote mesenchymal stem cell osteogenic differentiation; 5. Exhibits effective antibacterial activity against *Escherichia coli*; 6. Accelerates hydroxyapatite deposition in simulated body fluid (SBF)	1. Nanoparticle agglomeration prone to occur, impairing printing uniformity and structural integrity; 2. Difficult to precisely control ion release kinetics; 3. Long-term biosafety of degradation products requires further in-depth investigation	21–27
	β-TCP/HA/bioactive glass	PLA/β-TCP, PLA/HA, PLA/BG composites	1. Excellent osteoconductivity and bone-bonding ability; 2. Rapid induction of hydroxyapatite mineralization in SBF; 3. Reinforces mechanical properties of polymer scaffolds	1. High ceramic content increases filament brittleness and worsens printability; 2. Prone to structural defects during the 3D printing process	17, 28
Functional additive phases	Metal oxides (ZnO/SrHA)	Polymer/ZnO, polymer/SrHA composites	1. Synergistic antibacterial and osteogenic functions; 2. Bioactive ion release regulates local bone regeneration microenvironment	1. Potential cytotoxicity at high concentrations; 2. Poor dispersion uniformity in polymer matrix	29
	Antimicrobial peptides/antibiotics	Drug-loaded composite scaffold system	1. Targeted and efficient antibacterial effect; 2. Localized drug delivery reduces systemic side effects and antibiotic resistance risk	1. Poor stability of proteins and drugs during processing; 2. Obvious burst release effect difficult to control; 3. Complex preparation process	30–32
	Nanocarbon materials (GO/CNT)	Polymer/graphene oxide, polymer/carbon nanotube composites	1. Remarkably enhances scaffold mechanical strength; 2. Improves surface hydrophilicity and cell adhesion behavior	1. Controversial long-term biosafety and degradation profile; 2. High dispersion difficulty in polymer matrix	5
TPMS structural designs	Gyroid TPMS	Gyroid-type triply periodic minimal surface	1. High permeability and specific surface area; 2. Uniform stress distribution and excellent fatigue resistance; 3. Facilitates cell migration, nutrient transport, and waste exchange	Complex topological structure, demanding high printing accuracy and equipment performance	4, 33
	Diamond TPMS	Diamond-type triply periodic minimal surface	Excellent compressive mechanical strength and energy absorption capacity	Slightly lower pore interconnectivity compared with Gyroid TPMS	4, 34
	Primitive TPMS	Primitive-type triply periodic minimal surface	Regular structure, easy parameterized design, and modeling	Relatively weak mechanical properties and biological adaptability	4
	Gradient/multiscale TPMS	Multiscale/variable porosity TPMS structure	1. Mimics the heterogeneous structure of natural bone; 2. Balances mechanical support and vascular ingrowth requirements	High design and fabrication difficulty; low batch reproducibility	35, 36

## II. ADVANTAGES AND APPLICATIONS OF TPMS STRUCTURES IN THE DESIGN OF BONE TISSUE ENGINEERING SCAFFOLDS

### A. Basic characteristics and types of TPMS structures

Triply periodic minimal surfaces (TPMSs) are mathematically defined surfaces characterized by zero mean curvature and infinite periodicity in three dimensions. This unique geometric property results in smooth, continuous surfaces without sharp edges or corners, which is a critical advantage in reducing stress concentration points commonly observed in traditional porous structures.^37^ Among the most commonly studied TPMS types are the Gyroid, Diamond, and Primitive surfaces, each exhibiting distinct topological features yet sharing the fundamental minimal surface property. The smoothness and continuity of these surfaces facilitate uniform stress distribution under mechanical loads, thereby enhancing structural integrity and durability.^4^

A defining feature of TPMS structures is their highly interconnected porous network, which can be precisely controlled in terms of porosity and pore size through mathematical parameterization.^38^ This tunability is essential for biomedical applications, particularly bone tissue engineering, where pore architecture directly influences cell infiltration, vascularization, and nutrient-waste exchange. The interconnected pores promote efficient fluid flow and mass transport, which are vital for sustaining cell viability and promoting tissue regeneration. Compared to conventional regular porous structures such as cubic or cylindrical pores, TPMS architectures provide a more biomimetic environment that closely resembles the trabecular bone microstructure. This similarity extends not only to the mechanical response but also to the fluid dynamic environment, which is crucial for mimicking the physiological conditions of natural bone.^39^

The Gyroid surface, for example, is known for its complex yet smooth labyrinthine channels that facilitate high permeability and surface area, enhancing cell adhesion and proliferation.^33^ The Diamond and Primitive surfaces also offer unique pore geometries that can be exploited to tailor mechanical and biological properties. The ability to mathematically define and fabricate these structures using advanced additive manufacturing techniques such as stereolithography (SLA), fused deposition modeling (FDM), and laser powder bed fusion (LPBF) has accelerated their adoption in biomedical scaffold design.^20^ Furthermore, hierarchical TPMS structures, which combine multiple TPMS types or incorporate graded porosity, have been developed to further optimize mechanical strength and biological compatibility, demonstrating enhanced energy absorption and surface-to-volume ratios conducive to bone integration.

**FIG. 1.**
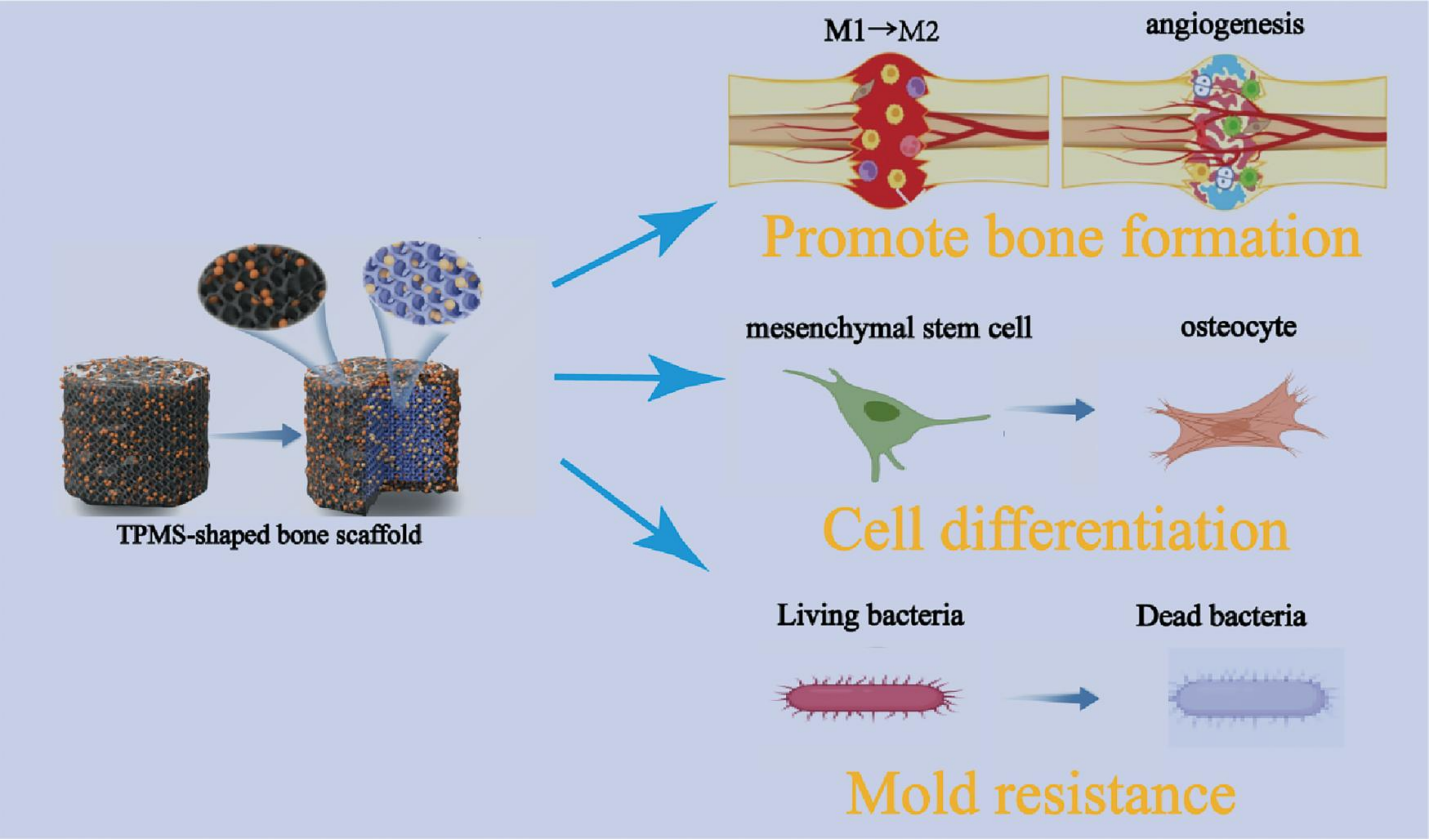
The 3D printing mechanism process of the TPMS structure bone scaffold.

In summary, TPMS structures are distinguished by their zero mean curvature, smooth continuous surfaces, and highly interconnected porous networks. Their mathematical parameterization allows precise control over porosity and pore size, enabling the design of scaffolds that closely mimic the mechanical and biological environment of natural bone.^40^ The common TPMS types—Gyroid, Diamond, and Primitive—each offer unique structural advantages, and their fabrication through additive manufacturing techniques has opened new avenues for creating advanced bone tissue engineering scaffolds with superior performance compared to traditional porous architectures (Fig. 1 and Table II).

**TABLE II.** Performance comparison of typical TPMS structures for bone scaffolds.

TPMS structure type	Mechanical properties (compressive strength/modulus)	Pore transport performance	Biological adaptability (cell and tissue response)	Optimal application scenario	References
Gyroid	Moderate strength and modulus, uniform stress distribution, low stress concentration	Ultra-high permeability and interconnectivity, smooth fluid flow, efficient nutrient/waste exchange	Superior cell adhesion, migration, and osteogenic differentiation; uniform cell distribution, easy vascular ingrowth	General critical-sized bone defects, non-load- to sub-load-bearing sites, infectious bone defects	4, 33, 41
Diamond	Highest compressive strength and energy absorption capacity, strong load-bearing capacity	Good interconnectivity, slightly lower permeability than Gyroid, stable fluid transport	Favorable cell adhesion, slightly slower cell infiltration than Gyroid, good osteoconductivity	Load-bearing bone defects (long bone, cortical bone repair), high mechanical demand scenarios	4, 25, 34
Primitive	Relatively low strength and modulus, poor resistance to compressive deformation	Regular pore structure, moderate permeability, simple mass transport	Basic cell compatibility, poor osteogenic induction effect, limited tissue integration	*In vitro* cell screening, preliminary material performance testing, non-load-bearing minor defects	4
Gradient/multiscale TPMS	Customizable gradient mechanical properties, matching natural bone heterogeneity, no stress shielding	Graded pore size distribution, regionally adjustable permeability, coordinated transport, and support	Synergistic effect of mechanical and physical cues, enhanced stem cell osteogenic differentiation, accelerated vascularization	Large segmental bone defects, complex irregular defects, clinical personalized bone repair	35, 36

### B. Optimization of scaffold mechanical and biological properties by TPMS structures

The intrinsic geometric features of TPMS structures confer significant advantages in optimizing both the mechanical and biological performance of bone tissue engineering scaffolds.^41^ Due to their minimal surface nature, TPMS architectures achieve high mechanical strength and toughness at relatively low material densities. This efficiency arises from the smooth, continuous curvature of the surfaces, which distributes stress uniformly and mitigates localized stress concentrations that typically lead to premature failure in porous scaffolds. For instance, the IWP (I-graph wrapped package) TPMS structure has demonstrated superior yield strength and energy absorption capacity compared to other TPMS types, attributed to its unique deformation mechanism characterized by layer-by-layer failure rather than shear banding.^34,42^ This mechanical robustness at reduced density is critical for matching the mechanical properties of scaffolds to those of different bone sites, thereby minimizing stress shielding and promoting natural load transfer.

Beyond mechanical strength, TPMS structures exhibit high specific surface areas due to their complex, continuous surfaces. This increased surface area enhances the interface between the scaffold material and biological environment, providing abundant sites for protein adsorption and cell adhesion. The enhanced cell–material interaction fosters improved cellular responses, including adhesion, proliferation, and differentiation, which are essential for effective tissue regeneration.^43^ Moreover, the smooth pore walls characteristic of TPMS reduce shear stress on cells during fluid flow, minimizing cell damage and guiding cell growth along the curved surfaces. This guidance promotes uniform cell distribution and organized tissue formation within the scaffold, as observed in studies where bone marrow stromal cells exhibited superior osteogenic differentiation on TPMS scaffolds compared to conventional grid structures.^44^

The tunability of TPMS structures also allows for the design of functionally graded scaffolds, where porosity and mechanical properties vary spatially to better replicate the heterogeneous nature of bone tissue. Such graded TPMS scaffolds have shown enhanced mechanical properties and energy absorption capabilities, as well as improved biological performance, by combining internal architectures inspired by trabecular bone with external structures mimicking cortical bone.^35^ Computational optimization methods, including topology optimization and machine learning approaches like backpropagation neural networks, have been employed to refine TPMS geometries for maximizing yield strength and permeability, further enhancing scaffold performance.^45^

Additionally, the interconnected porous network of TPMS scaffolds facilitates efficient nutrient and oxygen transport, as well as metabolic waste removal, which are critical for maintaining cell viability in three-dimensional constructs. Studies employing computational fluid dynamics and perfusion culture simulations have demonstrated that TPMS-based vascular-like flow channels optimize fluid flow and shear stress distribution, thereby improving *in vitro* culture conditions and potentially enhancing *in vivo* tissue integration.^46^ The permeability and wall shear stress within TPMS scaffolds can be finely tuned by adjusting structural parameters, enabling the creation of scaffolds that support active cell proliferation and vascularization.

In conclusion, TPMS structures optimize scaffold mechanical properties by enabling high strength and toughness at low densities through their smooth, continuous surfaces and unique deformation behaviors. Biologically, their high specific surface area and smooth pore walls enhance protein adsorption, cell adhesion, and proliferation, while their interconnected porous networks facilitate nutrient transport and vascularization. The ability to tailor these properties through geometric design and additive manufacturing positions TPMS-based scaffolds as highly promising candidates for bone tissue engineering applications (Table III).

**TABLE III.** 3D printing techniques for TPMS scaffolds: parameter optimization and fidelity comparison.

3D printing technique	Core optimized parameters	Structural fidelity	Minimum feature size (*μ*m)	Applicable materials for TPMS scaffolds	Suitable TPMS structure complexity	References
FFF/FDM	Nozzle temperature (180–220 °C); printing speed (20–60 mm/s); layer thickness (0.1–0.3 mm); infill density	Medium, prone to layer delamination and filament aggregation	200–300	Biodegradable polymers (PLA, PCL); polymer-ceramic composites	Medium, Gyroid, Primitive TPMS	16, 18, 19
SLA/DLP	UV light intensity; curing time; layer thickness (0.05–0.15 mm); resin viscosity	High, complete retention of complex curved surfaces	50–100	Photosensitive polymers; modified bioceramic resin	High, all TPMS types, especially gradient multiscale structures	20, 46
LPBF	Laser power; scanning speed; powder layer thickness; hatch spacing	High, suitable for dense metallic/ceramic structures	100–200	Titanium alloy; bioceramics (β-TCP, HA)	Medium-high, load-bearing Diamond TPMS	20, 34

## III. PRECISE MANUFACTURING OF TPMS STRUCTURE SCAFFOLDS VIA 3D PRINTING TECHNOLOGY

### A. Application of fused filament fabrication (FFF) technology in composite scaffold fabrication

Fused filament fabrication (FFF), also known as fused deposition modeling (FDM), is a widely adopted additive manufacturing technique that fabricates three-dimensional objects by the layer-by-layer deposition of molten thermoplastic filaments. This method is particularly suitable for processing polymers such as polylactic acid (PLA), owing to its relatively low melting temperature, biocompatibility, and biodegradability. The simplicity of the FFF process, combined with its cost-effectiveness, makes it an attractive choice for producing bone tissue engineering scaffolds with complex architectures, including triply periodic minimal surface (TPMS) structures.^16^ The core principle involves feeding a thermoplastic filament into a heated nozzle, where it is melted and extruded onto a build platform, solidifying upon cooling to form the desired geometry. This layer-wise approach enables precise control over scaffold porosity, pore size, and interconnectivity, which are critical parameters for facilitating cell infiltration, nutrient diffusion, and vascularization in bone regeneration applications.

To enhance the functional properties of scaffolds, FFF technology has been extended to fabricate polymer-ceramic composite filaments by pre-mixing PLA with bioactive ceramic powders such as magnesium titanate (MgTiO_3_), β-tricalcium phosphate (β-TCP), hydroxyapatite (HA), or bioactive glass (BG). The incorporation of ceramic fillers imparts osteoconductivity and mechanical reinforcement to the scaffolds, addressing the inherent limitations of pure polymers in bone tissue engineering. For instance, PLA/MgTiO_3_ composite filaments can be prepared by uniformly dispersing ceramic powders into the polymer matrix before filament extrusion, enabling the integrated printing of polymer-ceramic TPMS scaffolds with enhanced bioactivity and mechanical strength. This one-step fabrication approach ensures homogenous distribution of ceramic particles within the scaffold, preserving the intricate TPMS geometry and maintaining structural fidelity.^17^

The success of FFF in producing high-quality composite scaffolds hinges on the meticulous optimization of printing parameters, including nozzle temperature, printing speed, layer thickness, and infill density.^18^ Precise temperature control is essential to achieve uniform melting and extrusion of the composite filament, preventing nozzle clogging and ensuring consistent filament flow. Printing speed influences the bonding between layers and the resolution of fine features, while layer thickness affects surface roughness and mechanical anisotropy. For composite filaments, these parameters must be finely tuned to accommodate the altered rheological behavior caused by ceramic fillers, which can increase viscosity and affect melt flow. Studies have demonstrated that an optimal balance of these parameters leads to scaffolds with well-defined TPMS architectures, interconnected porosity, and mechanical properties compatible with native bone tissue. Moreover, the ability to fabricate composite scaffolds with tailored compositions and geometries via FFF facilitates the development of personalized implants for large bone defect repair, combining the biodegradability of PLA with the osteoinductive potential of ceramics.^1,19^

In summary, FFF technology offers a versatile and economical platform for the fabrication of polymer-ceramic composite scaffolds with complex TPMS structures. By integrating ceramic powders such as MgTiO_3_ into PLA filaments and optimizing printing parameters, it is possible to produce scaffolds that exhibit enhanced mechanical performance, bioactivity, and structural fidelity, thereby advancing the field of bone tissue engineering and personalized regenerative therapies.

### B. Correlation between printing accuracy, structural fidelity, and scaffold performance

Printing accuracy and structural fidelity are pivotal factors that directly influence the functional performance of TPMS-based bone tissue engineering scaffolds fabricated via FFF. Printing accuracy refers to the precision with which the printer can reproduce the designed geometry, including pore size, strut thickness, and overall dimensions, while structural fidelity denotes the degree to which the printed scaffold maintains the intended architectural features without deformation or defects. These parameters critically affect the scaffold's pore interconnectivity, surface roughness, and mechanical integrity, which in turn govern cellular responses and degradation behavior.^47^

High printing accuracy ensures that the designed TPMS pore structures are faithfully replicated, preserving the interconnected porous network essential for cell migration, nutrient transport, and vascularization. Precise control over pore size and distribution influences early cellular attachment and proliferation, as cells are sensitive to microenvironmental cues such as surface topography and pore geometry. For example, scaffolds with pore sizes in the range of 300–700 *μ*m have been shown to promote osteogenic differentiation and bone ingrowth. Deviations from the designed pore architecture due to printing inaccuracies can lead to pore blockage or irregularities, impeding cell infiltration and compromising scaffold functionality.

Structural fidelity is equally critical in maintaining the mechanical properties predicted by computational models. TPMS structures are designed to optimize mechanical strength while maximizing porosity; however, manufacturing defects such as layer delamination, incomplete fusion, or dimensional shrinkage can introduce local stress concentrations and weak points. These defects reduce the scaffold's load-bearing capacity and may accelerate degradation in localized regions, undermining the scaffold's ability to provide mechanical support during bone regeneration. Finite element analyses have demonstrated that scaffolds with high structural fidelity exhibit uniform stress distribution and mechanical resilience comparable to native trabecular bone.

Optimizing printing parameters is the core strategy to balance scaffold complexity, material printability, and functional performance. Parameters such as nozzle temperature, printing speed, layer height, and infill pattern must be tailored to the composite filament's rheological properties to achieve smooth extrusion and strong interlayer bonding. For instance, increasing nozzle temperature can improve filament flow but may degrade heat-sensitive components, while slower printing speeds enhance resolution but reduce manufacturing efficiency. Layer thickness influences surface roughness and mechanical anisotropy; thinner layers yield smoother surfaces and better feature resolution but increase print time. Additionally, the choice of infill pattern affects pore interconnectivity and mechanical behavior; TPMS geometries require precise deposition paths to maintain their minimal surface characteristics.

Recent studies have highlighted that the interplay between printing accuracy and structural fidelity significantly impacts the scaffold's biological performance. Scaffolds with well-preserved TPMS architectures facilitate early osteoblast adhesion and proliferation, promote uniform mineralization, and support sustained osteogenic differentiation. Moreover, consistent pore structures enable predictable degradation rates, ensuring gradual load transfer to regenerating bone and minimizing inflammatory responses. Conversely, scaffolds with compromised fidelity exhibit heterogeneous cell responses and unpredictable mechanical degradation, limiting their clinical applicability.

In conclusion, achieving high printing accuracy and structural fidelity is essential for fabricating TPMS scaffolds that meet the mechanical and biological demands of bone tissue engineering. Through systematic optimization of FFF printing parameters, it is possible to produce composite scaffolds with precise pore architectures, robust mechanical properties, and favorable cellular interactions, thereby enhancing their potential for effective bone regeneration.^15,48–51^

## IV. SYNERGISTIC ENHANCEMENT MECHANISM OF PLA/MgTiO_3_ COMPOSITES

### A. Synergistic enhancement of mechanical properties

The mechanical performance of bone tissue engineering scaffolds is a critical determinant of their clinical success, especially for load-bearing applications. Recent studies have demonstrated that incorporating MgTiO_3_ ceramic particles into a polylactic acid (PLA) matrix significantly enhances the mechanical properties of the resulting composite scaffolds. Specifically, the addition of MgTiO_3_ particles has been shown to increase the compressive strength by approximately 7.55% and the compressive modulus by 27.46%. This improvement is primarily attributed to the reinforcing effect of the ceramic particles and the efficient stress transfer between the PLA matrix and the MgTiO_3_ fillers. As rigid fillers, MgTiO_3_ particles restrict the mobility of PLA molecular chains, thereby increasing the stiffness of the composite. Under mechanical loading, these particles act as stress distributors, mitigating localized deformation and enhancing the overall load-bearing capacity of the scaffold. Such synergistic reinforcement brings the mechanical properties of PLA/MgTiO_3_ composites closer to those of cortical bone, which is essential for repairing bone defects in weight-bearing regions. This alignment with native bone mechanics not only supports physiological loads but also promotes favorable cellular responses by providing a mechanically stable environment conducive to osteogenesis. Moreover, the triply periodic minimal surface (TPMS) architectures commonly employed in 3D-printed scaffolds further optimize mechanical performance by balancing porosity and strength, as evidenced in studies comparing Diamond and Gyroid microarchitectures that exhibit superior compressive strength. The integration of MgTiO_3_ particles within such architecturally optimized scaffolds could thus synergistically enhance mechanical robustness while maintaining the interconnected porosity necessary for nutrient transport and tissue ingrowth. Overall, the strategic incorporation of MgTiO_3_ into PLA matrices represents a promising approach to engineer bone scaffolds with mechanical properties tailored to mimic natural bone, thereby addressing a key challenge in bone tissue engineering and expanding the applicability of 3D-printed scaffolds in clinical settings.^23–25^

### B. Improvement of thermal stability and hydrophilicity

Thermal stability and surface hydrophilicity are pivotal factors influencing the processing, structural integrity, and biological performance of bone tissue engineering scaffolds. The incorporation of MgTiO_3_ particles into PLA scaffolds has been shown to enhance thermal stability significantly, as evidenced by an increase in the thermal decomposition temperature from 320 to 338 °C. This elevation in thermal decomposition temperature suggests that MgTiO_3_ acts as a thermal barrier, delaying the onset of polymer degradation during processing and in physiological environments. Enhanced thermal stability is particularly advantageous during 3D printing processes such as fused deposition modeling (FDM), where elevated temperatures are employed, ensuring the scaffold maintains its structural integrity and dimensional accuracy. Furthermore, the presence of MgTiO_3_ modifies the surface properties of the composite scaffold, reducing the water contact angle from 94.2°, characteristic of hydrophobic pure PLA, to 76.8°, indicating increased hydrophilicity. This shift toward a more hydrophilic surface is critical for improving cell adhesion, spreading, and proliferation, as hydrophilic surfaces facilitate protein adsorption and subsequent cellular interactions. Enhanced hydrophilicity also promotes the infiltration of body fluids, which is essential for nutrient transport and waste removal within the scaffold microenvironment. These surface modifications contribute to improved biocompatibility and osteoconductivity, key attributes for successful bone regeneration. The synergistic effect of improved thermal stability and hydrophilicity thus not only facilitates scaffold fabrication and handling but also enhances the biological interface between the scaffold and host tissue. This dual enhancement aligns with findings in other composite systems where ceramic or nanoparticle incorporation improves both thermal and surface properties, thereby broadening the functional applicability of polymer-based scaffolds in bone tissue engineering^21,22^ (Table IV).

**TABLE IV.** Key functional indicators of PLA/MgTiO_3_ composite scaffolds.

Functional category	Performance indicator	Pure PLA group	PLA/MgTiO_3_ composite group	Performance improvement effect	References
Mechanical properties	Compressive strength Compressive modulus	Baseline value Baseline value	Increased by 7.55% Increased by 27.46%	Significantly enhanced mechanical robustness, matching sub-load-bearing bone requirements	23–25
Physical and thermal properties	Thermal decomposition temperature Water contact angle	320 °C 94.2° (hydrophobic)	338 °C 76.8° (hydrophilic)	Improved thermal stability for 3D printing; enhanced surface hydrophilicity for cell adhesion	21, 22
Bioactivity and osteogenesis	Hydroxyapatite deposition (SBF) MSC proliferation and osteogenic differentiation	Sparse and slow mineralization Basic cell activity, low osteogenic marker expression	Dense and rapid apatite formation Significantly enhanced proliferation, high ALP/osteocalcin expression	Excellent *in vitro* bioactivity, strong osteoinductive capacity via Mg^2+^ release	28, 52
Antibacterial property	Antibacterial rate against *E. coli*	No antibacterial activity	Significant bacteriostatic and bactericidal effect	Intrinsic antibacterial function, preventing implant-associated infection	26, 27

## V. BIOACTIVITY AND OSTEOINDUCTIVE PROPERTIES OF COMPOSITE TPMS SCAFFOLDS

### A. *In vitro* bioactivity and hydroxyapatite formation

The *in vitro* bioactivity of bone tissue engineering scaffolds is a critical determinant of their potential to integrate with host bone and promote regeneration. Immersing PLA/MgTiO_3_ triply periodic minimal surface (TPMS) scaffolds in simulated body fluid (SBF) for 24 days results in a significant increase in the formation of hydroxyapatite (HA) on their surfaces, underscoring the enhanced bioactivity conferred by the incorporation of MgTiO_3_. This enhancement is primarily attributed to the release of Mg^2+^ and TiO_3_^2−^ ions from MgTiO_3_ into the local microenvironment, which modulates the ionic concentration and pH, thereby facilitating the nucleation and growth of calcium phosphate crystals. The released Mg^2+^ ions are known to play a pivotal role in bone metabolism by stimulating osteoblast activity and mineralization, while TiO_3_^2−^ ions contribute to the stabilization of the apatite layer. The *in situ* formation of this bioactive HA layer is fundamental for establishing a robust chemical bond between the scaffold and the host bone, a process known as osseointegration. This bioactive interface not only enhances mechanical interlocking but also promotes cellular adhesion and proliferation, which are essential for subsequent bone tissue regeneration. Similar studies with composite scaffolds incorporating bioactive ceramics such as β-tricalcium phosphate (β-TCP) or mesoporous bioactive glass (MBG) have demonstrated rapid apatite crystallization within days of SBF immersion, confirming the critical role of bioactive components in accelerating HA deposition and scaffold bioactivity.^28^ Furthermore, the hierarchical porous architecture of TPMS scaffolds provides an optimal surface area and interconnected porosity that facilitate ion exchange and nutrient transport, further supporting apatite formation. The synergy between the TPMS structural design and the biochemical cues from MgTiO_3_ thus creates a conducive environment for biomimetic mineralization, which is indispensable for effective bone repair. Collectively, these findings highlight that the PLA/MgTiO_3_ TPMS scaffolds exhibit superior *in vitro* bioactivity through enhanced hydroxyapatite formation, laying a solid foundation for their application in bone tissue engineering.

### B. Promotion of mesenchymal stem cell proliferation and osteogenic differentiation

The biological performance of bone tissue engineering scaffolds is critically dependent on their ability to support the proliferation and osteogenic differentiation of mesenchymal stem cells (MSCs), which are key players in bone regeneration. *In vitro* cellular assays have demonstrated that the MgTiO_3_ component within PLA-based TPMS scaffolds significantly promotes the proliferation of human MSCs, indicating excellent cytocompatibility of the composite material. This proliferative effect is likely mediated by the bioactive ions released from MgTiO_3_, particularly Mg^2+^, which is known to activate multiple signaling pathways involved in cell growth and differentiation. Notably, Mg^2+^ ions can stimulate integrin-mediated signaling cascades, such as the focal adhesion kinase (FAK) and PI3K–Akt pathways, which regulate cytoskeletal organization, cell adhesion, and osteogenic gene expression. The activation of these pathways enhances the expression of osteogenic markers, including alkaline phosphatase (ALP), osteopontin, and osteocalcin, thereby promoting MSC differentiation toward the osteoblastic lineage. Importantly, the TPMS scaffold architecture provides a three-dimensional microenvironment that mimics the native extracellular matrix, offering physical cues such as optimal pore size, interconnectivity, and mechanical stiffness. This 3D microenvironment synergizes with the biochemical signals from MgTiO_3_, creating a dual induction system that fosters osteogenesis more effectively than either factor alone. Similar synergistic effects have been observed in composite scaffolds combining bioactive ceramics with polymeric matrices, where the structural and chemical cues collectively enhance MSC osteogenic differentiation.^41,52^ Moreover, the TPMS design ensures uniform cell distribution and nutrient diffusion, which are essential for sustained cell viability and function. The integration of MgTiO_3_ thus not only improves the scaffold's bioactivity but also actively modulates cellular behavior to accelerate bone tissue formation. These findings underscore the potential of PLA/MgTiO_3_ TPMS scaffolds as advanced biomaterials that harness both physical architecture and biochemical signaling to promote MSC proliferation and osteogenic differentiation, thereby advancing the field of bone tissue engineering.

## VI. ANTIBACTERIAL PROPERTIES AND MECHANISMS OF TPMS-STRUCTURED PLA/MgTiO_3_ SCAFFOLDS

### A. Antibacterial activity verification against *Escherichia coli*

The antibacterial efficacy of the PLA/MgTiO_3_ scaffold against *Escherichia coli* represents a pivotal advantage for its application as an anti-infective bone repair material. Studies have consistently demonstrated that incorporating metal oxides such as MgTiO_3_ into polymeric scaffolds significantly enhances their bactericidal properties, particularly against gram-negative bacteria like *E. coli*. The PLA/MgTiO_3_ composite scaffold exhibits strong antibacterial activity, which is critical in preventing post-implantation infections that can compromise bone healing and lead to implant failure. Quantitative and qualitative assessments of antibacterial performance are typically conducted using colony counting methods and live/dead staining assays. Colony counting provides a direct measure of bacterial viability by enumerating colony-forming units (CFUs) after exposure to the scaffold or its extracts, thereby quantifying the inhibition of bacterial proliferation. Live/dead staining, on the other hand, offers a visual and qualitative evaluation of bacterial membrane integrity and viability on the scaffold surface, distinguishing live bacteria from those compromised or killed by the material. These methodologies confirm that the PLA/MgTiO_3_ scaffold surface or its leachates effectively suppress *E. coli* growth, indicating a potent antibacterial environment. The interconnected porous architecture of the scaffold, fabricated via 3D printing, not only supports bone tissue ingrowth but also facilitates the interaction between antibacterial agents released from the scaffold and bacterial cells, enhancing the overall antimicrobial effect. This dual functionality—supporting osteogenesis while preventing bacterial colonization—addresses a critical clinical challenge in bone tissue engineering, where infection control is paramount for successful implant integration and bone regeneration.^26^

### B. Exploration of potential antibacterial mechanisms

The antibacterial mechanism of MgTiO_3_ within the PLA scaffold likely involves multiple synergistic pathways that disrupt bacterial viability and biofilm formation. One primary mechanism is the sustained release of Mg^2+^ ions from the MgTiO_3_ phase, which can interfere with bacterial cell membrane potential and enzymatic functions essential for bacterial metabolism and replication. Metal ions such as Mg^2+^ are known to destabilize bacterial membranes by altering electrochemical gradients and inhibiting key enzymes, leading to increased membrane permeability and eventual cell death. Additionally, the surface properties of the scaffold, particularly its hydrophilicity, are altered by the incorporation of MgTiO_3_, which may reduce the initial adhesion of bacteria and hinder biofilm establishment. Hydrophilic surfaces tend to resist bacterial attachment better than hydrophobic ones, thereby limiting the colonization and aggregation of bacterial cells on the scaffold. The triply periodic minimal surface (TPMS) structure of the scaffold further enhances antibacterial efficacy by providing a high specific surface area and a network of interconnected pores. This architecture facilitates the continuous and uniform release of antibacterial ions, ensuring sustained antimicrobial activity throughout the scaffold. Moreover, the porous network impedes bacterial settlement and biofilm maturation by disrupting the microenvironment necessary for bacterial aggregation. Importantly, the integration of antibacterial functionality with osteogenic potential in the PLA/MgTiO_3_ scaffold offers a comprehensive solution to bone defect repair. While the scaffold promotes bone regeneration through its biocompatible and osteoconductive properties, it simultaneously prevents implant-associated infections, a major clinical complication. This dual functionality not only improves the success rate of bone tissue engineering constructs but also reduces the reliance on systemic antibiotics, mitigating the risk of antibiotic resistance development. Thus, the PLA/MgTiO_3_ scaffold exemplifies an advanced biomaterial design that addresses both regenerative and anti-infective requirements in orthopedic applications^27,42^ (Table V).

**TABLE V.** Representative antibacterial strategies for TPMS-based bone scaffolds.

Antibacterial strategy	Typical antibacterial agents/systems	Antibacterial mechanism	Advantages	Limitations	Representative TPMS scaffold system	References
Inorganic ion antibacterial	Mg^2+^, Zn^2+^, Sr^2+^, Cu^2+^, MgTiO_3_, ZnO	Destroy bacterial cell membrane integrity; inhibit bacterial enzyme activity and metabolism; disturb intracellular ion balance	Good stability, sustained release effect; simultaneously promote osteogenesis; low cytotoxicity at appropriate concentration; no drug resistance risk	High concentration may cause cytotoxicity; ion release rate difficult to precisely control	PLA/MgTiO_3_ TPMS scaffold; ZnO/PCL Gyroid scaffold	26, 27, 29
Antibiotic loading	Vancomycin, gentamicin, amoxicillin	Inhibit bacterial cell wall synthesis or protein synthesis; targeted bactericidal effect	Strong immediate antibacterial effect; high bactericidal efficiency	Easy to induce bacterial resistance; burst release effect; poor thermal stability during 3D printing	Antibiotic-loaded gradient TPMS scaffold	30, 31
Antimicrobial peptides (AMPs)	Defensins, cathelicidins	Destroy bacterial membrane structure; broad-spectrum antibacterial effect	Low drug resistance risk; good biocompatibility	High cost; easy to degrade during processing; short half-life *in vivo*	AMPs modified Diamond TPMS scaffold	32
Photothermal/photodynamic therapy	Graphene oxide, MXene, black phosphorus	Generate local hyperthermia or reactive oxygen species to kill bacteria	Non-invasive, remote controllable; broad-spectrum antibacterial; promote osteogenesis simultaneously	Requires an external light source; potential local overheating damage	GO/PLA Gyroid TPMS photothermal scaffold	10, 53

## VII. APPLICATION PROSPECTS AND CHALLENGES OF MULTIFUNCTIONAL TPMS SCAFFOLDS IN BONE REPAIR

### A. Potential value in the repair of infectious bone defects

Infectious bone defects represent one of the most formidable challenges in orthopedic treatment due to the dual necessity of eradicating infection while simultaneously promoting effective bone regeneration. Traditional therapeutic approaches, primarily surgical debridement combined with prolonged systemic antibiotic administration, often fall short because they lack localized, sustained antibacterial activity and fail to provide an optimal microenvironment for bone healing. The development of multifunctional scaffolds that integrate structural biomimicry, mechanical support, osteoinductive capacity, and active antimicrobial properties is, therefore, critical. The PLA/MgTiO_3_ triply periodic minimal surface (TPMS) scaffold embodies such an integrated solution. Its TPMS architecture mimics the complex porous structure of natural bone, facilitating nutrient transport and cell infiltration while providing mechanical strength compatible with native bone tissue. The incorporation of MgTiO_3_ nanoparticles endows the scaffold with intrinsic antibacterial activity, addressing the critical need for local infection control without relying solely on systemic antibiotics. Moreover, the scaffold's biodegradability eliminates the necessity for secondary surgical removal, reducing patient morbidity and healthcare costs. During degradation, the scaffold releases Mg^2+^ ions, which have been shown to stimulate osteogenic differentiation and modulate the local microenvironment favorably, potentially enhancing long-term bone regeneration and remodeling. This sustained release of bioactive ions may also contribute to immunomodulation, further supporting tissue repair processes. Collectively, the PLA/MgTiO_3_ TPMS scaffold offers a promising one-stop platform for treating infectious bone defects by combining biomimetic design, mechanical integrity, osteoinduction, and active antimicrobial function, thereby overcoming the limitations of conventional materials and therapies.^16,54^

### B. Current challenges and limitations

Despite the promising attributes of PLA/MgTiO_3_ TPMS scaffolds, several critical challenges must be addressed to translate these materials into clinical practice effectively. One major issue is material homogeneity. Ensuring the uniform dispersion of MgTiO_3_ nanoparticles within the PLA matrix is essential to maintain consistent mechanical properties, degradation behavior, and antimicrobial efficacy across different production batches. Aggregation of nanoparticles can lead to localized stress concentrations, impair printability during 3D fabrication, and cause uneven ion release profiles, which may compromise scaffold performance and safety. Advanced mixing and fabrication techniques must be optimized to achieve nanoscale homogeneity and reproducibility. Another significant challenge lies in the degradation kinetics of the composite scaffold. The *in vivo* degradation rates of PLA and MgTiO_3_ must be carefully synchronized with the rate of new bone formation to provide sustained mechanical support without premature loss of structural integrity or excessive accumulation of degradation byproducts. An imbalance could result in scaffold collapse before sufficient bone regeneration or provoke inflammatory responses due to acidic or particulate degradation products. Comprehensive *in vitro* and *in vivo* studies are needed to elucidate the synergistic degradation mechanisms and to tailor scaffold composition accordingly. Furthermore, the long-term biological effects of MgTiO_3_ degradation products remain incompletely understood. While Mg^2+^ ions are generally considered osteogenic and biocompatible, the chronic exposure to MgTiO_3_ nanoparticles and their metabolites may elicit unforeseen cytotoxicity or immunogenicity, particularly at higher concentrations. The impact on local immune cells, including macrophage polarization and inflammatory signaling pathways, requires thorough investigation to ensure safety and efficacy. Additionally, potential systemic effects from nanoparticle dissemination must be evaluated through extended preclinical studies. Addressing these challenges through multidisciplinary research integrating materials science, biology, and engineering will be pivotal to harness the full therapeutic potential of PLA/MgTiO_3_ TPMS scaffolds for infectious bone defect repair^55,56^ (Table VI).

**TABLE VI.** Current challenges and future perspectives of multifunctional TPMS bone scaffolds.

Category	Current critical challenges	Corresponding improvement strategies	Future development trends	References
Material and composition	Nanoparticle agglomeration in composites; unbalanced degradation rate between matrix and filler; unclear long-term biosafety of inorganic fillers	Surface modification of fillers; optimize composite ratio; develop slow-release composite systems	Multi-component synergistic composite materials; intelligent responsive degradation materials; biomass-based green materials	29, 55, 56
Structure and manufacturing	Difficulty in balancing porosity and mechanical strength; low reproducibility of multiscale TPMS; high cost of high-precision printing	Computational topology optimization; multi-material gradient printing; parameter standardized control	4D bioprinting TPMS; multiscale hierarchical bionic structure; patient-customized integrated manufacturing	35, 36, 57
Function and mechanism	Uncontrollable ion/drug release; single function difficulty to meet clinical needs; unclear synergistic mechanism of antibacterial and osteogenesis	Micro/nano carrier loading; sequential controlled release system; in-depth mechanistic research at the cellular and molecular level	Multifunctional integrated scaffold; immunomodulatory combined bone repair; dynamic microenvironment responsive scaffolds	10, 30, 32
Clinical translation	Lack of standardized evaluation system; insufficient large animal experimental data; high clinical transformation cost; strict regulatory barriers	Establish unified *in vitro*/*in vivo* evaluation standards; carry out preclinical verification in large animals; optimize batch manufacturing process	Clinical grade material certification; minimally invasive implantable scaffolds; accelerated regulatory approval pathway	18, 58

## VIII. FUTURE RESEARCH DIRECTIONS AND DEVELOPMENT TRENDS

### A. Diversification and functional expansion of material systems

The advancement of bone tissue engineering scaffolds based on triply periodic minimal surface (TPMS) structures has increasingly focused on diversifying material systems and expanding their functionalities to address complex clinical challenges such as infection control and bone regeneration. One promising approach involves the integration of polylactic acid (PLA) with magnesium titanate (MgTiO_3_) and other bioactive substances, including growth factors and antimicrobial peptides, to construct drug delivery systems capable of sequential and controlled release. This strategy aims to achieve temporal multifunctionality, where an initial antibacterial effect is followed by enhanced osteogenesis, thereby addressing the dual demands of infection prevention and bone healing in infected bone defects. For instance, scaffolds incorporating vancomycin-loaded nanodiamond composites have demonstrated excellent antibacterial efficacy alongside osteogenic promotion, highlighting the potential of combining antimicrobial agents with osteoinductive components in a single scaffold platform.^30^ Similarly, the use of bioactive metal oxides such as zinc oxide (ZnO) and strontium-containing hydroxyapatite (SrHA) has been explored to endow scaffolds with ion release profiles that not only inhibit bacterial colonization but also stimulate osteoblast proliferation and differentiation. Surface functionalization techniques, such as seed-assisted hydrothermal growth of ZnO nanoarrays on polycaprolactone (PCL) scaffolds, have shown enhanced osteogenic activity and early differentiation of osteoblast-like cells, indicating the feasibility of integrating metal oxide nanostructures with TPMS architectures for multifunctional applications.^29^ Beyond metal oxides, the incorporation of bioglass and other bioactive ceramics into polymer matrices like PLA has yielded composite scaffolds with improved mechanical strength and bioactivity, as demonstrated by PLA–bioglass composites that exhibit homogeneous particle distribution and favorable *in vivo* biocompatibility. The exploration of other metal oxides or bioactive glasses with distinct ionic release spectra and functional emphases could lead to a new family of materials tailored for specific clinical scenarios, such as enhanced angiogenesis or immunomodulation. Moreover, the combination of these materials with advanced drug delivery systems, including microsphere-based carriers and nanodiamond composites, facilitates spatiotemporal control over therapeutic agent release, which is critical for managing infection and promoting bone regeneration in a staged manner.^31,32^ The integration of growth factors, antimicrobial peptides, and ions within TPMS scaffolds thus represents a multifaceted approach to scaffold functionalization, enabling the design of next-generation bone tissue engineering constructs that can dynamically respond to the biological environment and clinical needs.

### B. Innovations in manufacturing technology and multiscale structural design

The manufacturing of TPMS-based bone tissue engineering scaffolds has witnessed significant innovations, particularly through the integration of multi-material 3D printing technologies and the design of multiscale hierarchical structures that better mimic the heterogeneity of native bone tissue. Multi-material 3D printing enables the fabrication of scaffolds with spatially varied material compositions and porosity gradients within a single construct, thereby replicating the complex mechanical and biological microenvironments of bone. This gradient design approach allows for the optimization of scaffold regions to fulfill distinct functions, such as enhanced mechanical support in load-bearing zones and increased porosity for vascularization and cell infiltration in regenerative zones.^36^ The ability to engineer such gradients within TPMS architectures is particularly advantageous, as the continuous and smooth surfaces of TPMS structures facilitate controlled variation in pore size and interconnectivity, which are critical for nutrient transport and osteointegration. Furthermore, advancements in high-resolution printing techniques, such as digital light processing (DLP), have enabled the production of TPMS scaffolds with feature sizes at the micro- and nanoscale. These finer topological features are instrumental in modulating cell behavior, including adhesion, proliferation, and differentiation, through precise control of the scaffold's microenvironment. For example, the incorporation of conductive carbon nanotube coatings via facile one-step functionalization on 3D-printed scaffolds has been shown to enhance pre-osteoblast adhesion and osteogenic gene expression, especially when combined with electrical stimulation, underscoring the importance of nanoscale surface modifications. Additionally, cryogenic 3D printing techniques have been employed to fabricate nanofibrous scaffolds embedded with antibacterial nanoparticles, such as nanosilver and zinc-coated black phosphorus, which provide multifunctional properties including infection resistance and osteogenic promotion.^53,57^ The convergence of these manufacturing innovations with TPMS design principles facilitates the creation of scaffolds that not only possess biomimetic mechanical properties but also exhibit tailored biological functionalities at multiple scales. This multiscale structural design, coupled with precise material deposition, holds promise for advancing scaffold performance in clinical bone defect repair by better recapitulating the native bone microenvironment and enhancing cell–scaffold interactions (Table VII).

**TABLE VII.** Comparative analysis of TPMS scaffolds and traditional porous scaffolds. TPMS, triply periodic minimal surface; FFF, fused filament fabrication; FDM, fused deposition modeling; SLA, stereolithography; DLP, digital light processing; LPBF, laser powder bed fusion; PLA, polylactic acid; MgTiO_3_, magnesium titanate; β-TCP, β-tricalcium phosphate; HA, hydroxyapatite; PCL, polycaprolactone; GO, graphene oxide; MSC, mesenchymal stem cell; ALP, alkaline phosphatase; SBF, simulated body fluid; AMPs, antimicrobial peptides.

Evaluation indicator	TPMS structure scaffolds	Traditional porous scaffolds (cubic/cylindrical pore)	Core superiority of TPMS	References
Structural feature	Zero mean curvature, smooth continuous surface, no sharp corners, 3D periodic	Regular geometric pores, sharp edges and corners, simple structure	No stress concentration points, uniform mechanical distribution	4, 37
Mechanical property	High strength and toughness at low density, adjustable mechanical gradient, no stress shielding	Uneven stress distribution, easy to break at edges, poor toughness	Better matching with natural bone mechanics, higher structural stability	34, 41
Mass transport performance	High interconnectivity, smooth fluid flow, efficient nutrient and waste exchange	Poor pore connectivity, easy to form dead space, low fluid transmission efficiency	More conducive to cell survival, proliferation and vascular ingrowth	33, 46
Biological response	Promote uniform cell distribution, reduce cell shear damage, enhance osteogenic differentiation	Uneven cell adhesion, easy to accumulate locally, low osteogenic efficiency	Better bionic microenvironment, stronger bone regeneration ability	43, 44

### C. Standardized evaluation systems and clinical translation pathways

The translation of multifunctional composite scaffolds based on TPMS architectures from bench to bedside necessitates the establishment of standardized evaluation systems that comprehensively assess their dual antibacterial and osteogenic functionalities. Current research underscores the importance of developing *in vitro* and *in vivo* models that dynamically evaluate the scaffold's performance in resisting infection while promoting bone regeneration. For instance, antibiotic-eluting collagen–hydroxyapatite scaffolds with dual-release kinetics have been assessed in murine and rabbit models of osteomyelitis, demonstrating effective bacterial eradication alongside facilitated bone healing, thereby exemplifying the need for integrated evaluation of antimicrobial efficacy and osteoconductivity. To systematically characterize such multifunctional scaffolds, standardized protocols encompassing microbiological assays, cytocompatibility tests, osteogenic differentiation markers, and histological analyses are essential. Moreover, dynamic assessment models that simulate the temporal progression of infection and bone repair can provide more clinically relevant insights into scaffold performance. Beyond laboratory evaluation, large animal models that closely mimic human infected bone defect scenarios are critical for validating scaffold safety and efficacy prior to clinical trials. For example, patient-specific medical-grade polycaprolactone-tricalcium phosphate (mPCL-TCP) scaffolds combined with autologous bone grafts and growth factors have been successfully implanted in segmental long bone defects in large animals, showing promising bone regeneration and integration.^18,58^ Such preclinical studies are indispensable for addressing challenges related to scaffold mechanical stability, biodegradation, immune response, and infection control in a physiological context. Furthermore, the development of regulatory frameworks and manufacturing standards tailored to multifunctional 3D-printed scaffolds will facilitate their clinical translation. This includes quality control of material composition, reproducibility of scaffold architecture, and validation of drug release profiles. Collectively, the establishment of robust, standardized evaluation systems and well-designed translational pathways will accelerate the clinical adoption of TPMS-based multifunctional scaffolds, ultimately improving therapeutic outcomes for patients with infected bone defects.

## IX. CONCLUSION

The development of 3D-printed PLA/MgTiO_3_ composite scaffolds based on TPMS (triply periodic minimal surface) structures represents a significant advancement in bone tissue engineering, merging sophisticated structural design with innovative material composition to address multifaceted clinical challenges. From an expert perspective, this approach exemplifies how the integration of topology and bioactive materials can synergistically enhance scaffold performance, balancing mechanical robustness with biological functionality—a balance that is critical for successful bone regeneration.

TPMS architectures inherently provide an optimized porous network that mimics the natural trabecular bone microenvironment, offering superior mechanical efficiency and facilitating nutrient transport and cell infiltration. This structural design is pivotal in achieving mechanical properties that can withstand physiological loads while maintaining a conducive environment for cellular activities. The incorporation of MgTiO_3_ into the PLA matrix introduces bioactive and antibacterial properties that are essential for promoting osteogenesis and preventing post-implantation infections, which remain significant hurdles in bone repair. MgTiO_3_ enhances hydrophilicity, thereby improving cell adhesion and proliferation, and promotes hydroxyapatite (HA) deposition, which is crucial for bone mineralization. Additionally, its antibacterial effects help mitigate infection risks, a common complication in bone defect treatments.

The interplay between TPMS topology and MgTiO_3_'s bioactivity creates a multifunctional scaffold capable of supporting stem cell differentiation toward osteogenic lineages while simultaneously inhibiting bacterial colonization. This dual functionality addresses the often conflicting demands of mechanical integrity and biological activity in scaffold design. However, despite promising preclinical results demonstrating feasibility and efficacy, several challenges remain before clinical translation can be realized. Achieving material homogeneity within the composite is essential to ensure consistent mechanical and biological performance. Furthermore, controlling the degradation rate of the scaffold to match new bone formation is critical to avoid premature loss of mechanical support or prolonged presence that could impede tissue remodeling.

Long-term biocompatibility and biosafety must be rigorously evaluated, considering the potential cytotoxicity or inflammatory responses associated with degradation products. Additionally, scaling up manufacturing processes while maintaining precision and reproducibility poses a significant technical challenge. Advances in additive manufacturing technologies and quality control protocols will be necessary to produce these complex scaffolds at a clinically relevant scale.

Looking forward, the future of TPMS-based multifunctional bone scaffolds lies in continued material innovation, such as incorporating other bioactive ceramics or growth factors to further enhance osteoinductivity and angiogenesis. Refinements in 3D printing techniques, including multi-material printing and real-time monitoring, will improve scaffold customization and functional integration. Moreover, establishing standardized evaluation criteria for mechanical properties, degradation behavior, and biological performance will facilitate comparative studies and regulatory approval processes.

In conclusion, the convergence of TPMS structural design and MgTiO_3_ bioactive composite materials offers a promising platform for developing next-generation bone repair scaffolds. By effectively balancing mechanical strength, osteogenic potential, and antibacterial activity, this strategy addresses critical limitations of current bone graft substitutes. With ongoing research focused on overcoming translational barriers, these multifunctional scaffolds have the potential to revolutionize the treatment of complex bone defects, particularly those complicated by infection, ultimately improving patient outcomes and advancing the field of regenerative medicine.

## Data Availability

The data that support the findings of this study are available from the corresponding author upon reasonable request.
